# Enhanced sensitivity in THz plasmonic sensors with silver nanowires

**DOI:** 10.1038/s41598-018-33617-2

**Published:** 2018-10-19

**Authors:** J. T. Hong, S. W. Jun, S. H. Cha, J. Y. Park, S. Lee, G. A. Shin, Y. H. Ahn

**Affiliations:** 1 Department of Physics and Department of Energy Systems Research, Ajou University, Suwon, 16499 Korea; 2 Department of Environmental Engineering, Ajou University, Suwon, 16499 South Korea

## Abstract

We developed hybrid slot antenna structures for microbial sensing in the THz frequency range, where silver nanowires (AgNWs) were employed to increase the sensitivity. In order to fabricate the hybrid devices, we partially etched the AgNW in the slot antenna region, where we can expect the field enhancement effect at the AgNW tip. We measured the resonant-frequency shift observed upon the deposition of a polymer layer, and observed that the sensitivity increased upon the introduction of AgNWs, with an enhancement factor of more than four times (approximately six times in terms of figure-of-merit). The sensitivity increased with the AgNW density until saturation. In addition, we tested devices with PRD1 viruses, and obtained an enhancement factor of 3.4 for a slot antenna width of 3 μm. Furthermore, we performed finite-difference time-domain simulations, which confirmed the experimental results. The sensitivity enhancement factor decreased with the decrease of the slot width, consistent with the experimental findings. Two-dimensional mapping of the electric field confirmed the strong field localization and enhancement at the AgNW tips.

## Introduction

Terahertz (THz) spectroscopy is an efficient technique for non-contact, non-destructive, and label-free investigations of biological and chemical substances^1–6^. However, it is challenging to detect microorganisms such as fungi, bacteria, and viruses, as their scattering cross-sections are significantly smaller than the THz wavelengths^7^. We recently developed highly sensitive microbial sensors for the detection of the microorganisms in the THz frequency range, using plasmonic and metamaterial devices, based on the sensitivity of their resonant frequency to changes in the dielectric constant of the slot area^7–9^. In particular, the plasmonic devices that consist of periodically arranged sub-wavelength rectangular holes exhibit excellent electromagnetic properties including shape resonance^10^. The resonant transmission in slot antenna structures is associated with extremely localized and enhanced fields that enable sensitive sensing of conducting or dielectric materials^8,11,12^.

In order to optimize the device sensitivity, the effects of geometrical factors, such as the width of the antenna and substrate refractive index, on the sensitivity have been investigated. For instance, it is crucial to reduce the effective refractive index at the gap area for a better sensitivity, which can be achieved using substrates with a low refractive index and virtually free-standing films^8,9^. In particular, the sensitivity significantly increases with the decrease in the gap width owing to the enhanced field localization^8,11,13–16^. More recently, nanogaps were incorporated in the metamaterials using the nanolithography method for an enhanced detection of viruses with a typical size smaller than 100 nm^15^. However, it is expensive and time-consuming to fabricate such nanoscale structures using conventional lithographic methods; advanced techniques are required to achieve the localized fields. Therefore, it is desirable to incorporate novel functional materials that have an inherent nanoscale dimension such as one-dimensional nanowires.

Recently, network films consisting of nanomaterials such as single-walled nanotubes (SWNTs), graphene, reduced graphene oxide, and silver nanowires (AgNWs) have emerged as a potential platform for optoelectronic devices^17–22^. Solution-based processes allow us to use relatively low-cost methods of spin-coating, roll-to-roll processing, and printing. AgNWs exhibit superior electrical and optical properties, which make them of interest for applications in THz optoelectronic devices. Recently, we demonstrated that AgNW network films can be employed in plasmonic devices in the THz frequency range^23^; however, biological sensing applications with an enhanced sensitivity were not addressed. In addition, the relatively thick AgNW networks were fabricated as an alternative to conventional metallic films, and the effect of the nanoscale morphology of the individual AgNWs was not addressed.

In this study, we fabricated THz hybrid slot antenna devices with individual AgNWs protruding in the slot area, for a highly sensitive detection of polymer films and microorganisms such as viruses. We observed a resonant-frequency shift (Δ*f*) in the shape resonance upon the depositions of poly(methyl methacrylate) (PMMA) and viruses. Here, a nanoscale detection volume and enhanced sensitivity were achieved without using the nanolithography approach. The sensitivity was investigated as a function of the density of the AgNWs and slot antenna width. The experimental results were confirmed by finite-difference time-domain (FDTD) simulations.

## Results and Discussion

**Figure 1:**
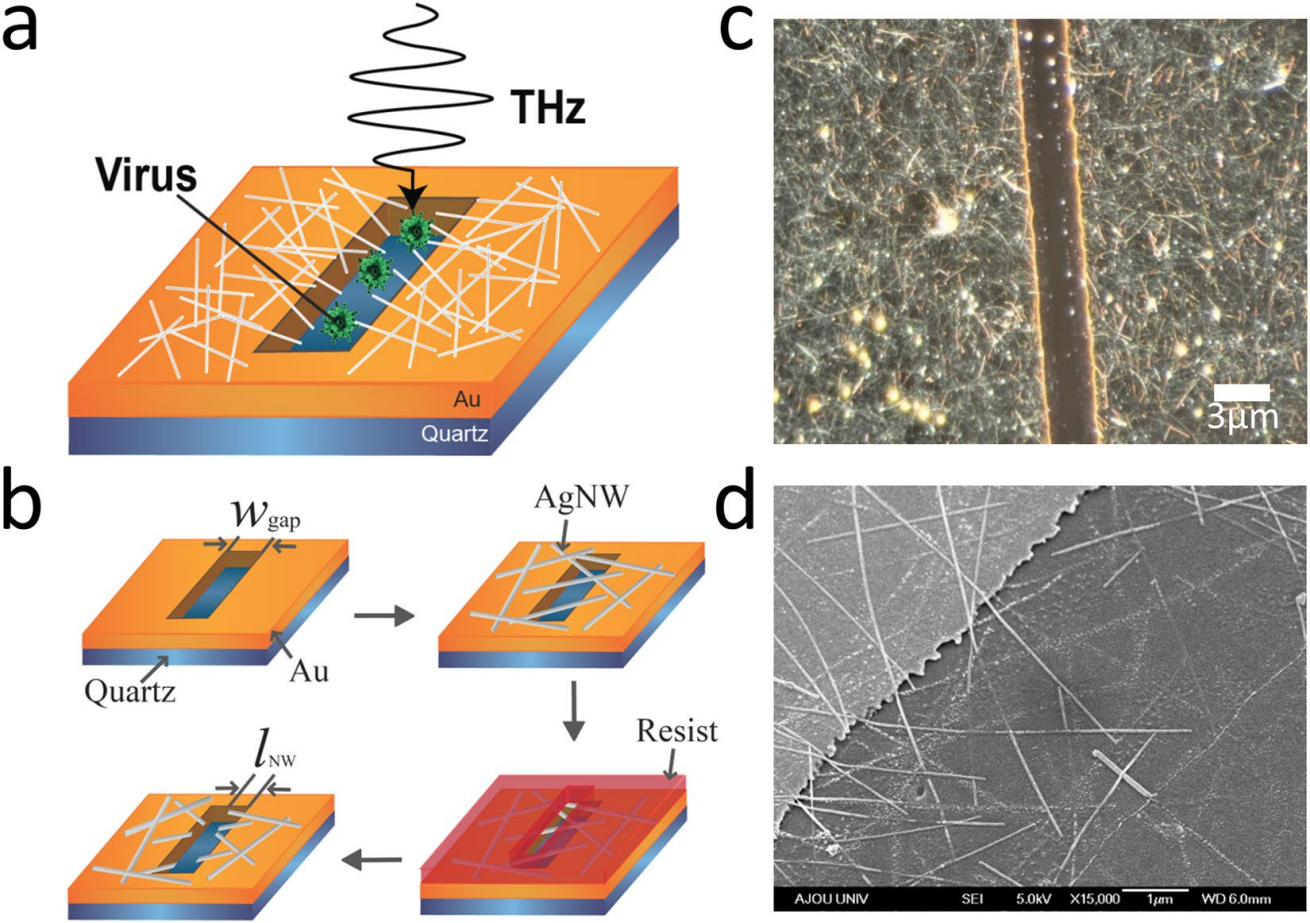
(**a**) Schematic of the THz hybrid slot antenna with protruding AgNWs. (**b**) Schematic of the fabricating processes for the THz hybrid slot antenna. (**c**) Optical image of the rectangular slot antenna with AgNWs (scale-bar: 3 μm). (**d**) SEM image near an edge of the slot antenna with AgNWs protruding towards the slot area.

The experiment is illustrated in Fig. 1(a). We fabricated conventional THz slot antenna devices, except that there are additional AgNWs protruding towards the slot region. We expect an additional field enhancement mediated by the sharp edges of the AgNWs to achieve an enhanced sensitivity for sensing of viruses. The THz hybrid plasmonic devices were fabricated using a two-step process. First, we prepared conventional THz plasmonic devices with lengths (*L*) of and various widths (*w*_gap_) and coated a AgNW film on the slot antenna devices by spin-coating a AgNW dispersion solution. In order to remove a part of the AgNW film, we performed another photolithography process, followed by etching with Al etchant. The average diameter and length of the individual AgNWs used for the network film were approximately 20 nm and 20 μm, respectively. Throughout the experiments, the length of the protruding part (*l*_NW_) on both sides of the aperture was kept at *l*_NW_ = *w*_gap_/3 (therefore, *l*_NW_ is in the range of 1–5 μm), as illustrated in Fig. 1(b). Representative optical and scanning electron microscopy (SEM) images of the hybrid devices are shown in Fig. 1(c,d), respectively. For the biological sensing experiments, viruses (PRD1) with a size of 60 nm were prepared, as described in a previous study^24^.

**Figure 2:**
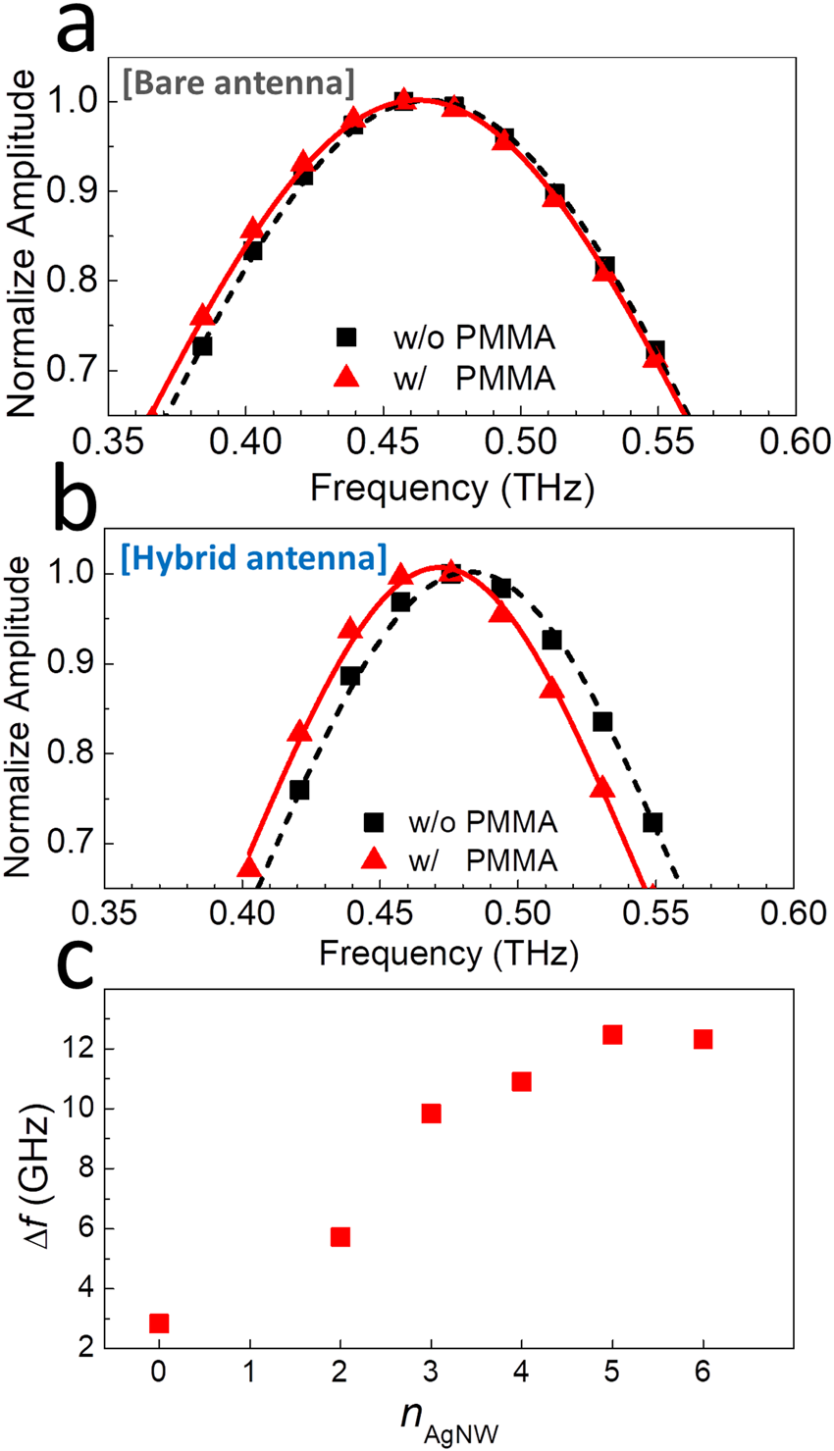
Normalized transmission amplitudes through the (**a**) bare slot antenna (*L* = 200 μm and *w*_gap_ = 15 μm) and (**b**) hybrid slot antenna (*l*_NW_ = 5 μm) with (red) and without (black) PMMA. (**c**) Resonant-frequency shift upon the deposition of PMMA as a function of the AgNW spin-coating time (*n*_AgNW_) using the solution of 0.15 g/ml.

The real-time THz transmission amplitudes of the hybrid plasmonic devices were measured using a conventional THz time-domain spectroscopy (TDS) setup^7^. First, we sense the polymer films (PMMA) with the hybrid sensors, as shown in Fig. 2. Figure 2(a) shows the normalized THz transmission with (red) and without (black) the PMMA coating on the slot antenna sensors without the AgNW hybrid structures. For the bare device with a length of *L* = 200 μm and width *w*_gap_ of 15 μm, a resonance peak is observed near 0.46 THz before the deposition of the polymer film on the device. The resonance frequency is primarily determined by the length of the antenna, i.e., *f*_0_ = *c*/2*n*_eff_*L*, where *n*_eff_ is the effective refractive index, determined by the refractive indices of the air and substrate, as reported in previous studies^25^. When the polymer film was coated with a thickness of 1 μm, a slight red-shift by approximately 2.8 GHz was observed in the resonance frequency.

In contrast, for the hybrid device (with *l*_NW_ = 5 μm), a considerable red-shift by 10 GHz was observed in the resonant frequency, as shown in Fig. 2(b), which is an increase of approximately 3.8 times, with respect to the bare slot antenna devices. The hybrid device were fabricated by spin-coating the AgNW solution (0.15 g/ml) three-times on the bare device, followed by the chemical etching process. The significant enhancement in the response can be attributed to the field enhancement effect induced by the protruded metal NWs in the gap area, referred to as the lightening effect. In addition, the resonant frequency (*f*_0_) without the deposition of PMMA changes by approximately 15 GHz with the introduction of the AgNW structures; furthermore, the quality factor (*Q*) increased from 2.2 to 3.5. This demonstrates the advantage of using AgNW hybrid structures, which improve the figure-of-merit (FOM) approximately 5.4 times (from 0.014 to 0.075); FOM is defined as FOM = *QS*/*f*_0_, where *S* = Δ*f*/*n*_film_ is the sensitivity (*n*_film_ is the refractive index of the target film)^26^.

In order to confirm the effect of the AgNWs on the enhanced sensitivity, Δ*f* was studied for various densities of the AgNWs. Figure 2(c) shows Δ*f* as a function of the number of AgNW coatings (*n*_AgNW_) using the same AgNW solution as in Fig. 2(b). Roughly 50 AgNWs were found at each side of the slot edge (when *L* = 200 μm) for *n*_AgNW_ = 1, whereas their length and orientation are randomly distributed. Δ*f* rapidly increased with *n*_AgNW_, from Δ*f* = 2.83 for *n*_AgNW_ = 0 (i.e., without the AgNWs) to Δ*f* = 12.3 for *n*_AgNW_ = 6. The sensitivity improved by more than 4.3 times in terms of Δ*f* (6.7 times in terms of FOM).

**Figure 3:**
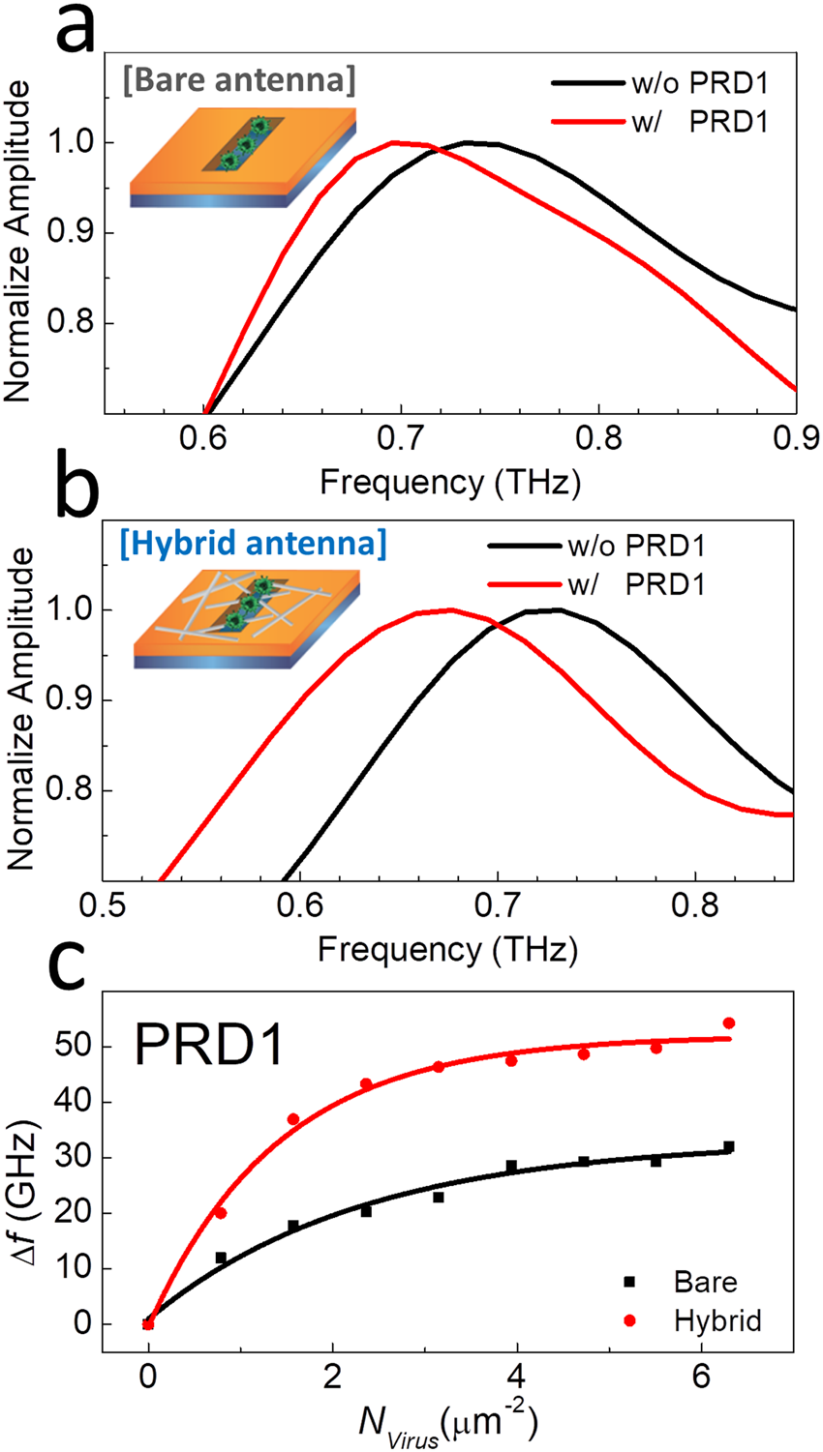
Normalized transmission amplitudes through the (**a**) bare slot antenna (*L* = 100 μm and *w*_gap_ = 3 μm) and (**b**) hybrid slot antenna (*l*_NW_ = 1 μm) with (red) and without (black) PRD1 viruses. (**c**) Resonant-frequency shift as a function of the surface number density (*N*_virus_) of viruses for the hybrid slot antenna (red) and bare slot antenna (black).

As mentioned above, THz plasmonic and metamaterial devices are an effective platform for the sensing of microorganisms such as fungi and bacteria^7,8,15^. In particular, the sensitivity improved by an order of magnitude by introducing nanogaps in the metamaterials for sensing viruses; however, this requires to use the nanolithography approach to obtain the nanoscale structures, which is not efficient for a large-scale production of the devices. Therefore, the proposed novel hybrid devices could be a very efficient platform for a highly sensitive microbial detection. In this study, we tested the proposed hybrid sensors using the representative virus PRD1, as shown in Fig. 3. Figure 3(a) shows the THz transmission amplitudes before and after the coating of PRD1 with a density of 6.3 μm^−2^. The length and width of the slot antenna were 100 μm and 3 μm, respectively. The resonant frequency shifted by Δ*f* = 32.0 GHz upon the deposition of the PRD1 viruses. For the hybrid antenna devices (*l*_NW_ = 1 μm and *w*_gap_ = 3 μm), Δ*f* significantly increased to 54.2 GHz, approximately 1.7 times larger than that of the bare device at the same virus surface density, as shown in Fig. 3(b). Figure 3(c) shows Δ*f* as a function of the PRD1 number density when they are deposited on the hybrid (red) and bare (black) slot antenna devices.

The frequency shift exhibits saturation behavior for both samples; the curves can be fitted with the relation Δ*f* = Δ*f*_sat_(1 − exp(−*N*_virus_/*N*_sat_)), where Δ*f*_sat_ is the saturation frequency, *N*_virus_ is the number density of PRD1, and *N*_sat_ is the saturation number density. For a dielectric sensing, GHz/refractive-index-unit-(RIU) is a common unit to represent the sensitivity; however, the sensitivity for the low-density-virus detection can be better represented by the frequency shift for a given surface density. The sensitivity (*S*_virus_) can be defined by the initial slope of the curves in Fig. 3(c), leading to *S*_virus_ = Δ*f*/*N*_virus_^15^. The sensitivity in terms of number of virus particles increases from 12.8 GHz·μm^2^/particle (bare antenna) to 32.7 GHz·μm^2^/particle (hybrid antenna), which is an increase of 2.5 times (3.4 times in terms of FOM, considering the increase of the *Q*-factor of 1.29 times).

Because the THz measurement process takes only a couple of seconds, improving the sensitivity will be the key element toward practical applications. The sensitivity of our hybrid devices allows us to identify the viruses with the surface densities lower than 0.1 unit/μm^2^. We want to point out here that we specify the sensitivity in terms of the surface density, whereas the volume density is commonly adopted in sensing with aqueous solution. Accordingly, the sensitivity of the hybrid sensors will be also influenced by the drop-casting times. In other words, the better sensitivity can be achieved if we deposit the analytes more times from the given solution density. Given that we performed the single drop-casting, our sensitivity corresponds to as low as ~10^7^ unit/mL, and obviously, the sensitivity could reach as low as ~ 10^7^ *N*_drop_^−1^ unit/mL, where *N*_drop_ is the drop-casting times. For instance, 10^6^ unit/mL can be reached for *N*_drop_ = 10, which will add 30 min to the measurement time. The sensitivity has to be improved further for the practical applications, because currently available assays are capable of detecting plasma viruses at a detection limit of 1,000 unit/mL, whereas some of the possible applications with the detection limit of ~10^6^ unit/mL can be found in the early diagnosis of disease and the inspection of rivers^27–29^.

**Figure 4:**
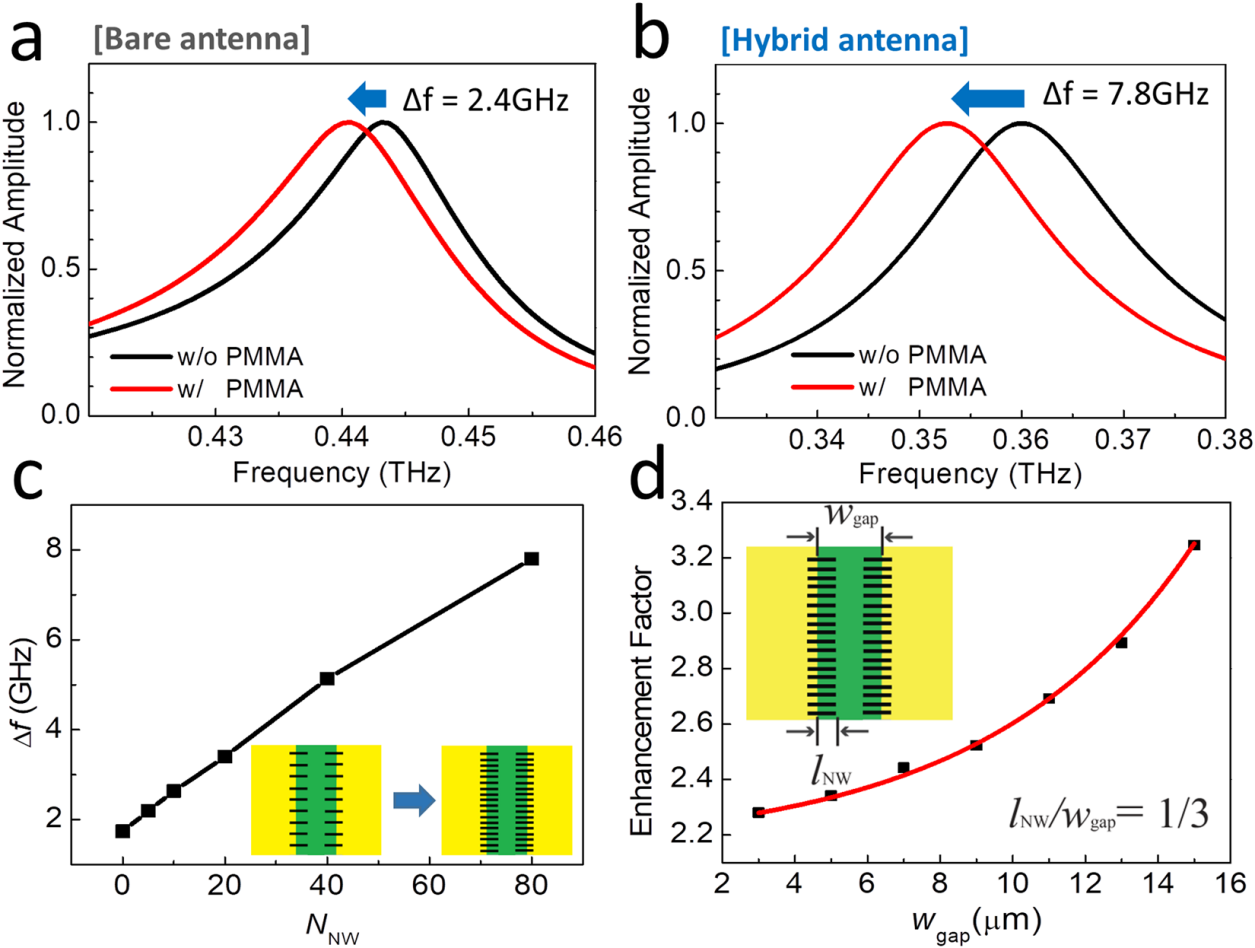
(**a**) FDTD simulation results of transmission spectra through the bare slot antenna with (red) and without (black) PMMA, for *L* = 200 µm and *w*_gap_ = 15 µm. (**b**) Transmission spectra through the hybrid slot antenna with (red) and without (black) PMMA for *l*_NW_ = 5 µm. (**c**) Resonant-frequency shift as a function of the total number of AgNWs (*N*_NW_). (**d**) Enhancement factor as a function of *w*_gap_ for fixed *N*_NW_ = 80 and *l*_NW_/*w*_gap_ = 1/3. (The solid line is a guide to the eye.).

Further, FDTD simulations were performed to reproduce our results. The Hybrid sensor was modeled using a CST simulation to imitate the electromagnetic responses in our experiments. We employed a linearly polarized plane wave as a source and periodic boundary conditions to simulate an array of the slot antenna structure. As shown in Fig. 4, we used slot antenna patterns with geometric parameters similar with those employed in the experiments (width and height of 20 nm); the metal film for the bare slot antenna was considered a perfect electric conductor. In order to reproduce the effects of the polymer films, we added a 1-μm-thick dielectric film on the slot antenna structure. The dielectric constant of the film was set to *ε*_f_ = 2.56, equal with that of the PMMA film.

As shown in the normalized spectra in Fig. 4(a), for the bare slot antenna devices (*L* = 200 μm and *w*_gap_ = 15 μm), the resonant frequency of the THz slot antenna exhibits a red-shift of 2.4 GHz with the introduction of the dielectric film. Figure 4(b) shows that the hybrid antenna device exhibits a higher frequency shift of 7.8 GHz, where a total number (*N*_NW_) of 80 AgNWs (*l*_NW_ = 5 μm) are present. The sensitivity increased by approximately 3.25 times, consistent with the results shown in Fig. 2. Figure 4(c) shows Δ*f* as a function of *N*_NW_ in the slot antenna with *L* = 200 μm and *w*_gap_ = 15 μm. It increases with the number density of AgNWs, as observed experimentally in Fig. 2(c). In addition, the sensitivity shows increasing tendency with *l*_NW_ until it shows a saturation behavior (Supplementary Information Figure S1). We also investigated the sensitivity enhancement factor as a function of the width of the bare slot antenna as shown in Fig. 4(d), while maintaining the relative ratio between the NW length and antenna width at *l*_NW_/*w*_gap_ = 1/3. The enhancement factor increases with the bare slot antenna width, consistent with the experimental findings, which showed a larger sensitivity enhancement for the results in Fig. 2, compared with those in Fig. 3.

**Figure 5:**
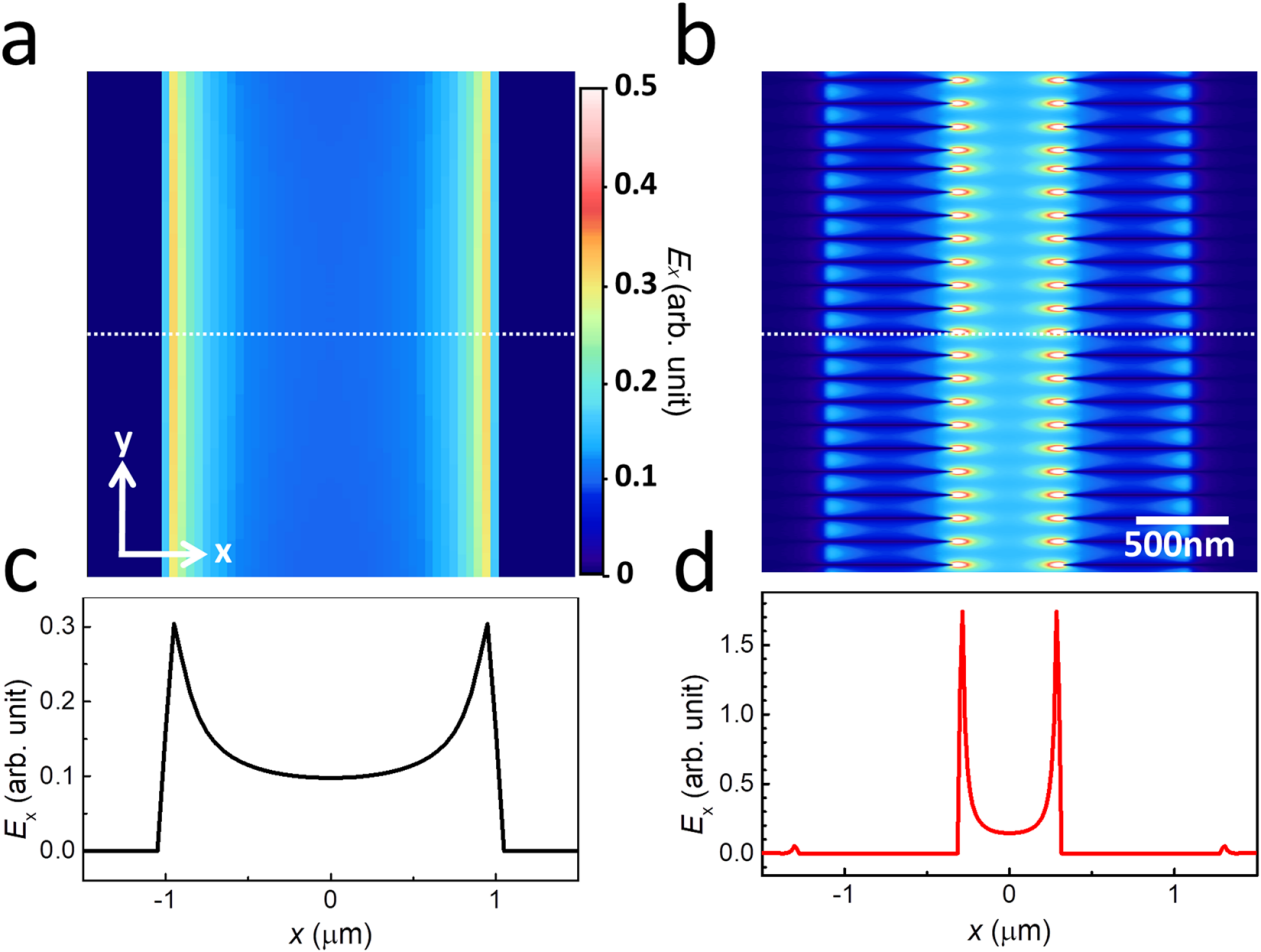
(**a**) Two-dimensional (2D) mapping of the electric-field (*E*_x_) distribution around the slot antenna area on the substrate surface, obtained from the FDTD simulation. (**b**) 2D electric-field distribution (*E*_x_) for the hybrid slot antenna devices. The height and width of the AgNW were 30 nm; (**c**) and (**d**) line profiles of *E*_x_ along the dotted lines in (**a**) and (**b**), respectively.

To provide further insight into the mechanisms of enhanced sensitivity, we show the electric-field distribution (*E*_x_) around the gap area in Fig. 5(a,b), for the bare and hybrid structures, respectively, with *L* = 45 μm and *w*_gap_ = 2 μm. Unlike in the bare antenna device, we could clearly identify strong spots at the ends of the individual NWs, indicating the large field enhancement, 5.91 times larger than that in the bare antenna device. Importantly, the total electric field (integrated over the entire slot area) increases with the addition of AgNWs by about 3.1 time, owing to the strong field localization along the z-axis. This is consistent with the NW-induced, sensitivity enhancement factor discussed so far, and explain why we could achieve the enhanced sensitivity even when the analytes are randomly distributed over the entire surface. We also note that the improved sensitivity do not originate from the gap-narrowing effects owing to the increased coverage of metallic region. In other words, we found that the sensitivity is even higher for the hybrid structure, when compared with the bare device having the narrow width (Supplementary Information Figure S2). Therefore, the additional field-enhancement near the tip end plays a crucial role. The extremely localized detection volume (~27 × 45 × 73 nm^3^), determined by the size of the NWs, is very useful for the sensing of viruses, as it matches with the size of the viruses (Supplementary Information Figure S3). Therefore, it is highly desirable to develop a novel device fabrication step to localize the target substances exclusively near the NW’s ends.

We also point out that the terahertz metamaterial sensors will operate very efficiently in aqueous environment. This is because the detection volume, which is highly confined near the surface, allows us to use very thin water layer (20 μm) free from the large attenuation effect^7,30^. Furthermore, the effective detection volume of the hybrid sensor is even more localized near the surface (~70 nm in the vertical range) due to the AgNW morphology. Therefore, our approach will constitute an important step toward fabricating the highly sensitive biosensors, enabling the high-speed on-site detection of live and viable microorganisms in various environments.

## Conclusion

We developed hybrid slot antenna structures for microbial sensing in the THz frequency range, where the sensitivity was increased with the introduction of the AgNWs. In order to fabricate the hybrid devices, we etched the AgNW partially in the slot antenna region, and we could expect the field enhancement effect at the NW tip. We measured the resonant-frequency shift with the deposition of a polymer layer, and observed that the sensitivity increased for the device with AgNWs, with an enhancement factor of more than 3.8 (~6.7 in terms of FOM) for the slot antenna width of 15 μm. The sensitivity increased with the AgNW density until saturation. In addition, we tested the devices using PRD1 viruses, and obtained an enhancement factor of 2.5 (~3.4 in terms of FOM) for a slot antenna width of 3 μm. We performed FDTD simulations, which were consistent with the experimental results. The sensitivity enhancement factor decreased with the decrease of the slot width, consistent with the experimental findings. The 2D mapping of the electric field confirmed the strong field localization and enhancement at the AgNW tips. We were able to achieve a nanoscale detection volume accompanied by the strong field enhancement without employing nanoscale fabrication methods. This study contributes to the development of novel efficient sensors, for various applications including the highly sensitive microbial detection, with an enhanced sensitivity achieved using novel functional materials.

## Methods

### Terahertz time-domain spectroscopy

The real-time THz transmission amplitudes of the hybrid devices were measured using a conventional THz time-domain spectroscopy (TDS) setup^7^. A femtosecond laser (*λ* = 800 nm) was incident on the photoconductive antenna, which radiated a linearly polarized THz pulse. The pulse was focused on the slot antenna array with a spot area of ~1 mm^2^ under ambient conditions. Time traces of the transmitted THz electric field, for both amplitude and phase, were measured by varying the time delay between the probe beam and THz pulse. The THz spectrum is obtained by applying a fast Fourier transform (FFT) to the time trace and normalized with respect to the reference.

### Fabrication of Hybrid Slot antenna Patterns

We prepared conventional THz plasmonic devices on quartz substrates using the conventional photolithography method, followed by a metal deposition of Cr/Au (2 nm/78 nm) using a thermal evaporator. The slot antenna arrays consist of slot antenna patterns with lengths (*L*) of 100 μm and 200 μm, and various widths (*w*_gap_) in the range of 3–15 μm. We coated a AgNW film on the slot antenna devices by spin-coating a AgNW dispersion solution (with a weight percentage of 0.15 g/ml). The speed and duration of the spin-coating were 800 rpm and 20 s, respectively. The average diameter and length of the individual AgNWs used for the network film were approximately 20 nm and 20 μm, respectively. In order to remove a part of the AgNW film, we performed another photolithography process, followed by etching with Al etchant (Aluminum Etchant Type A) for 1 s.

### Deposition of PMMA and PRD1

To test the sensitivity of the hybrid devices, we spin-coated a PMMA layer (6% in Chlorobenzene, molecular weight ${{\rm{M}}}_{{\rm{w}}}$ = 950,000 $g/\mathrm{mol}$). The speed and duration of the spin-coating were 3000 rpm and 40 s, respectively. PRD1 viruses were prepared by the double agar layer plaque technique^24^. We deposited the PRD1 solution onto hybrid devices, followed by drying in an 80 °C hot plate for 3 min. The drying process was adopted to reduce the preparation time by evaporating the water surrounding the viruses without losing their unique characteristics.

### Finite Difference Time-Domain (FDTD) Simulation

To predict the electromagnetic behavior of the Hybrid devices, the structure is simulated by using CST MICROWAVE STUDIO.

## Electronic supplementary material

Supplementary Information
