# Supplementary material for: Enhanced sensitivity in THz plasmonic sensors with silver nanowires
Source: Sci Rep. 2018 Oct 19;8:15536. doi: 10.1038/s41598-018-33617-2 (PMC6195569; doi:10.1038/s41598-018-33617-2)
Supplement: Supplementary file 1 — Supplementary Information [file 41598_2018_33617_MOESM1_ESM.doc]

**Supplementary Information**

Enhanced sensitivity in THz plasmonic sensors with silver nanowires

*J. T. Hong^1^, S. W. Jun^1^, S. H. Cha^1^, J. Y. Park^1^, S. Lee^1^, G. A. Shin^2^ and Y. H. Ahn^1^,**

*^1^Department of Physics and Department of Energy Systems Research, Ajou University, Suwon 16499, Korea*

*^2^Department of Environmental Engineering, Ajou University, Suwon 16499, South Korea*

**S1. Frequency shift as a function of NW length**


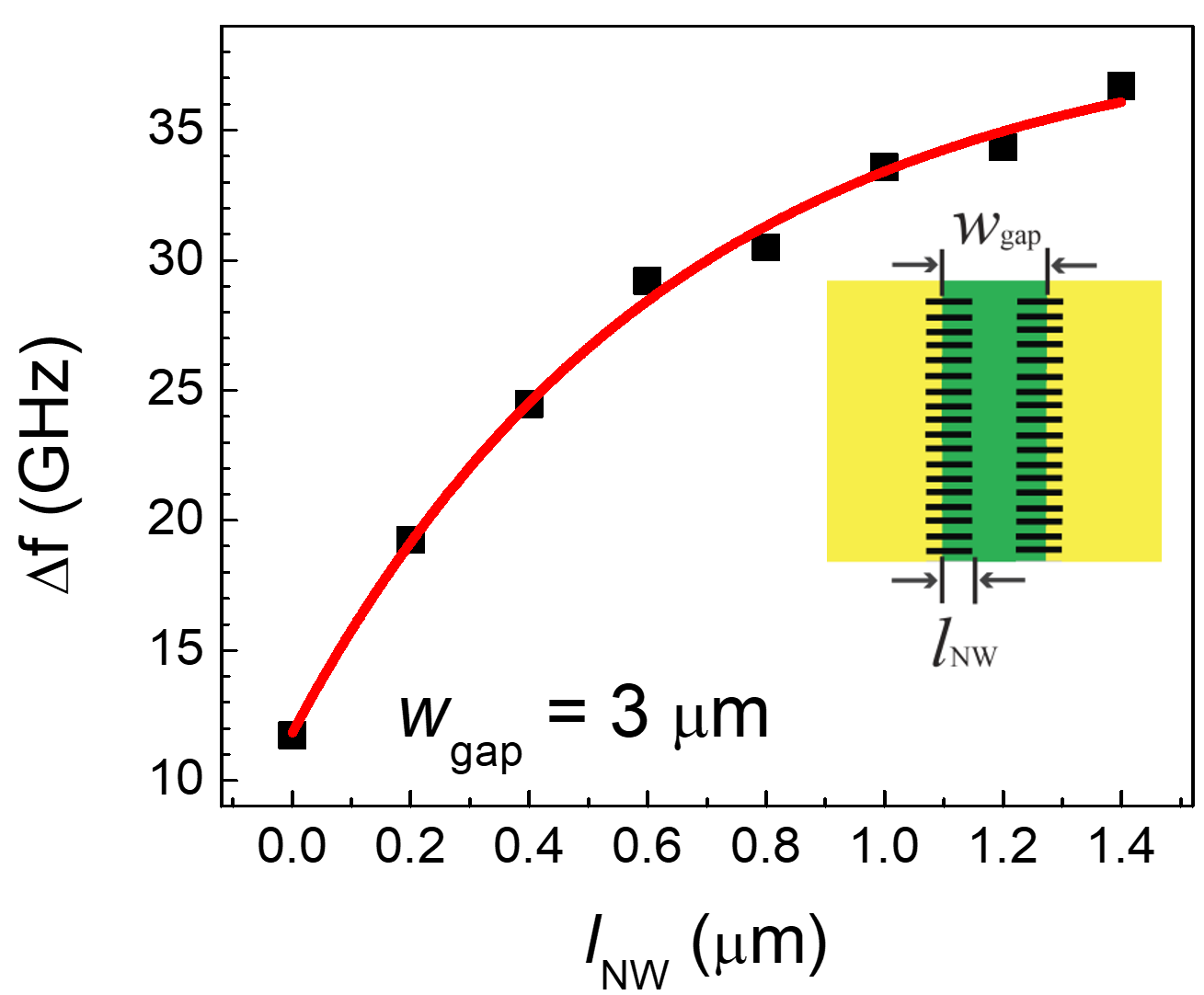


**Figure S1.** The frequency shift (Δ*f*) of the hybrid slot antenna in the presence of PMMA layer were plotted as a function of Ag nanowire length (*l*_NW_) from the FDTD simulation results. The width and length of the slot were fixed at *w*_gap_ = 3 μm and *L* = 100 μm, respectively. The frequency shift increases with *l*_NW_ initially, whereas it shows a saturation behavior for larger *l*_NW_. In our experimental condition of *l*_NW_/*w*_gap_ = 3, Δ*f* reaches more than 90 % of the maximum frequency shift.

**S2. Sensitivity of the hybrid sensors in comparison with a narrow-gap device**


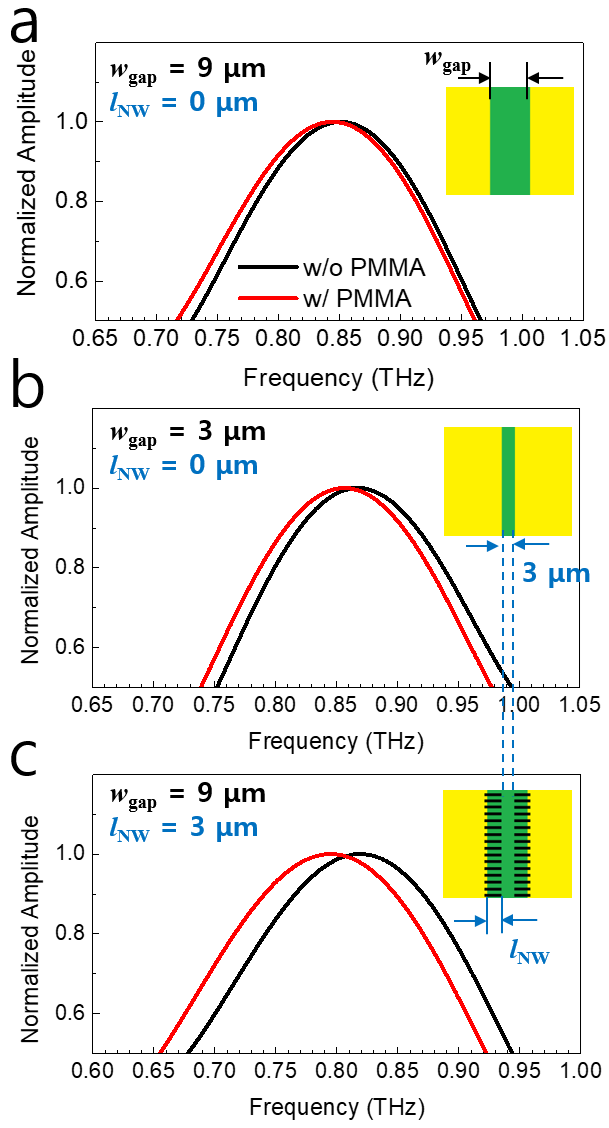


**Figure S2.** (a) Transmission amplitudes of a wide-gap slot antenna (*w*_gap_ = 9 μm) with and without the PMMA layer, obtained from the FDTD simulation. (b) Transmission amplitudes of a narrow-gap slot antenna (*w*_gap_ = 3 μm). (c) Transmission amplitudes of a hybrid slot antenna with a wide gap of *w*_gap_ = 9 μm and the AgNW length of *l*_NW_ = 3 μm. The frequency shifts were measured at 6.7 GHz, 11.7 GHz, and 24.0, respectively, for (a), (b), and (c). This result implies that the sensitivity is higher for the hybrid structures than the bare devices with a narrow gap, even when the metal-free region of the hybrid structure in (c) is comparable to that of (b). Therefore, the sensitivity enhancement in the hybrid structures cannot be determined simply by the gap-narrowing effects imposed by the metallic NWs.

**S3. 2D mapping of electric fields transverse to the NW axis**


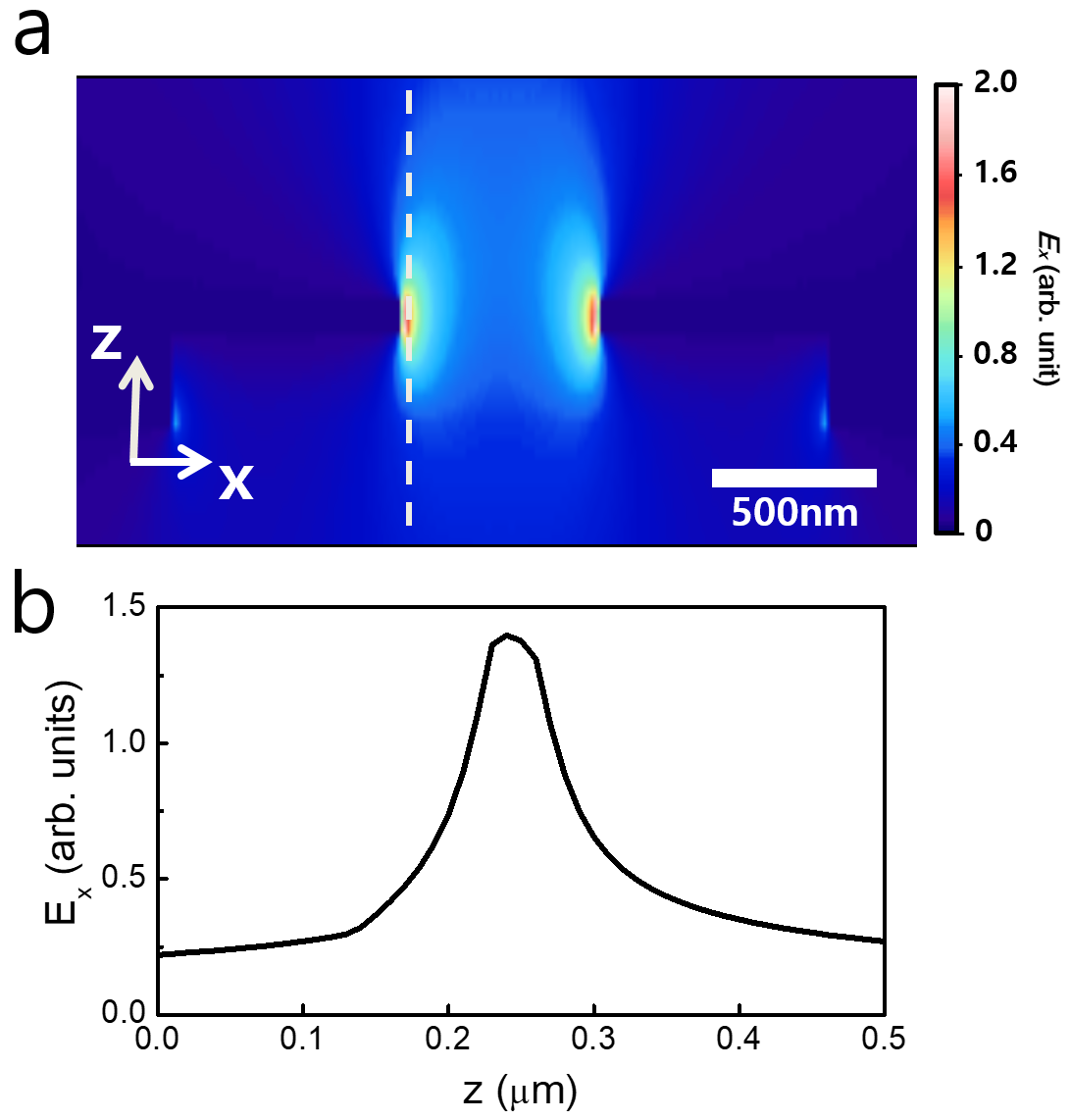


**Figure S3.** (a) Two-dimensional (2D) mapping of the electric-field (*E*_x_) distribution along *x*-*z* plane around the slot antenna area, obtained from the FDTD simulation. (b) A line profile of *E*_x_ along the dashed lines in (a). We measured the vertical range of the detection volume at the tip of NWs (i.e., along the z-axis), which was found to be 73 nm in term of full-width at half-maximum.
